# Potential for Improved Glycemic Control with Dietary *Momordica charantia* in Patients with Insulin Resistance and Pre-Diabetes

**DOI:** 10.3390/ijerph110202328

**Published:** 2014-02-21

**Authors:** Jimmy T. Efird, Yuk Ming Choi, Stephen W. Davies, Sanjay Mehra, Ethan J. Anderson, Lalage A. Katunga

**Affiliations:** 1 East Carolina Heart Institute and the Center for Health Disparities, Brody School of Medicine, East Carolina University, Greenville, NC 27834, USA; 2 Adult Gerontological Nurse Practitioner Program, School of Nursing, University of North Carolina at Greensboro, Greensboro, NC 27402, USA; E-Mail: y_choi6@uncg.edu; 3 Department of General Surgery, University of Virginia School of Medicine, Charlottesville, VA 22908, USA; E-Mail: sd2wf@virginia.edu; 4 Department of Cardiovascular Sciences, Brody School of Medicine, East Carolina University, Greenville, NC 27834, USA; E-Mail: mehras@ecu.edu; 5 Department of Pharmacology and Toxicology, Brody School of Medicine, East Carolina University, Greenville, NC 27834, USA; E-Mails: andersonet@ecu.edu (E.J.A.); katungal10@students.ecu.edu (L.A.K.); 6 East Carolina Diabetes and Obesity Institute (ECDOI) at East Carolina University, 115 Heart Drive, East Carolina Heart Institute, Greenville, NC 27834, USA

**Keywords:** *Momordica charantia*, bitter melon, insulin resistance, pre-diabetes, glycemic control

## Abstract

Bitter Melon (*Momordica charantia)* is a widely used traditional remedy for hyperglycemia. While the medicinal properties of this plant have been studied extensively using *in vitro* and animal models, the clinical efficacy and safety in humans is largely unknown. This review discusses the benefits and limitations of bitter melon supplementation in the context of epidemic levels of insulin resistance and pre-diabetes throughout the world.

## 1. Introduction

An estimated 79 million adult Americans have pre-diabetes, a condition that is preventable and treatable if recognized early [1]. Patients presenting with pre-diabetes have glycated hemoglobin (HgA1c) and blood sugar levels that are below the clinical threshold to be classified as type 2 diabetes but are higher than normal (HgA1c 5.7–6.4%; fasting blood glucose 100–125 mg/dL; 2-hour plasma glucose with 75 g oral glucose tolerance test between 140–199) [2]. However, the early stages of damage to the heart and circulatory system have already begun and without intervention, pre-diabetics are at risk for becoming diabetic and developing cardiovascular disease [3,4]. While the percentage of pre-diabetes is equal among blacks and whites (35%), a disproportionate percentage of non-Hispanic blacks progress to diabetes compared with their non-Hispanic white counterparts (18.7% *vs*. 10.2%) [1]. Guidelines established by the American Association of Clinical Endrocrinologists (AACE) suggest that regular glucose monitoring and adoption of healthful lifestyles (diet and exercise) may reverse uncomplicated pre-diabetes [5]. 

Nearly 40% of Americans use complementary and alternative medicine (CAM) for health promotion and the treatment of illness [6]. Among this group, between 2 and 3 million specifically use CAM in an attempt to lower blood sugar levels and to treat various stages of diabetes, despite limited studies of their safety and efficacy [7]. CAM is most commonly used by immigrant groups and their descendants, including Asians, Latin Americans, Africans, Indians, West Indians, and Native Americans [7]. However, CAM use is not limited to these groups and many Americans have become increasingly aware of CAM practices from their immigrant and Native American neighbors. Because of the rapidly changing demographics of the United States and the wide use of CAM, primary care providers need to be knowledgeable about CAM in order to appropriately advise their patients regarding the safety and efficacy of these practices. For example, potentially harmful interactions with conventional medications may occur if an effective CAM therapy is added to conventional therapy [7]. Furthermore, some patients, especially among vulnerable populations, may not disclose CAM use to their primary care provider because of historic mistrust of the health care system [8]. Many CAM therapies are based on traditional remedies and have not been evaluated under rigorous scientific conditions (e.g., large-scale, placebo-controlled, randomized clinical trials using standardized protocols) [7]. Nonetheless, 25% of prescription drugs on the market today contain active compounds derived from plants or plant-derived synthetic analogs (e.g., metformin and the French lilac plant) [9]. Approximately 80% of the world’s population routinely depends on plant derived therapies to prevent and treat illness [9].

Previous animal studies have observed an increase in insulin secretion and glucose uptake with the use of some herbal compounds [10]. However, it remains important to carefully evaluate the potential effectiveness and safety of these compounds in controlling blood sugar levels in humans. Evidence of the therapeutic benefits of folkloric medications cannot be presumed solely from their historical use or data extracted from animal studies alone. Below, we review the clinical evidence to date pertaining to *Momordica charantia* (bitter melon), a traditional herbal remedy used since ancient times by Chinese and Ayurvedic practitioners to treat high blood sugar and early signs of diabetes [11,12].

## 2. Description

Bitter melon is a commonly consumed vegetable that is found throughout the sub-tropical world (China, India, Thailand, East Africa, The Caribbean, Central and South America) and is known by various names, such as balsam pear, bitter gourd, carilla, cerasee (wild variety), cundeamor, goo-fah, and karela [13,14]. The plant has been well studied in animal models and several mechanisms of action have been proposed for its anti-diabetic properties (Table 1). At latest count, approximately 228 different compounds with possible medicinal properties, acting alone or in combination, have been isolated from bitter melon fruit, seeds, leaves, stems, pericaps, endosperm, callus tissues, and cotyledons [15]. Among these, the most actively studied constituents shown to improve glycemic control include charatin, polypeptide-p, vicine, momordin, and similar derivatives (e.g., momordinol, momordicilin, momorcharin, momordicin) [15,16,17].

**Table 1:** Antidiabetic mechanisms of bitter melon (*Momordica charantia*) in animal models.

Mode of Action	Model	Observed Effects
**1. Insulin signaling pathway**	▪ *db/db* mice ▪ Whole fruit powder, lipid, saponin or the hydrophilic residue of bitter melon fruit ▪ 5 weeks	▪ ↓ Hba1c 10% ▪ ↓ Protein tyrosine phosphatase 1B (PTP 1B) activity by 20% [18]
	▪ Non- obese diabetic mice ▪ Recombinant ADMc1 (active ingredient isolated from Bitter Melon)	▪ ↓ Glucose after 8 h intraperitoneal injection [19]
	▪ C57Bl6/J mice, high fat diet ▪ Bitter melon fruit ▪ 16 weeks	▪ ↑ Liver tyrosine phosphorylation ▪ ↑ Insulin receptor substrate 1 ▪ ↓ Plasma apoB-100 and apoB-48 in HFD-fed mice [20]
	▪ C57Bl6/J mice, high fat diet ▪ Bitter melon, aquatic extract powder ▪ 12 weeks	In skeletal muscle: ▪ ↑ Insulin receptor substrate-1 (IRS-2) ▪ ↑ Insulin receptor β (IRβ) ▪ ↑ Phosphatidylinositide 3-kinases (PI 3-K) ▪ ↑ Glucose transporter type 4 (GLUT4) ▪ ↑ Phosphorylation of insulin receptor substrate-1, Akt1 and Akt2 ▪ ↓ Body weight, plasma glucose, insulin, leptin levels, and HOMA-IR [21]
**2. Gluconeogenic & Glycolysis enzymes**	▪ Rats ▪ Bitter melon fruits extracts	▪ ↓ Hexokinase activity and glucose uptake activity in intestinal fragments [22]
	▪ Streptozotocin treated rats ▪ Bitter melon leaf extract ▪ 90 min	▪ ↓ Activity ~30% Hepatic glucose-6-phosphatase and fructose 1,6-bisphosphatase activities [23]
	▪ C57Bl6/J, high fat diet ▪ Bitter melon fruit extract ▪ 4 weeks	▪ ↑ mRNA and protein expression of glucose transporter 4 (GLUT4) ▪ ↓ Hyperglycemia, hyperleptinemia, HbA1c and free fatty acid ▪ ↑ Adipose PPARγ and liver PPARγ mRNA levels [24]
**3. Pancreatic Beta cells**	▪ Streptozotocin Wistar rats ▪ Bitter melon fruit pulp ▪ 28 days	▪ ↑ Fasting blood, glucose, serum insulin and cell function ▪ ↑ Diabetic rat cells were abundant with insulin granules ▪ ↑ Islet size and total cell areaNumber of β-cells ▪ ↑2 fold with bitter melon treatment [25]
	▪ Alloxan treated albino rats ▪ Bitter melon fruit ▪ 15 and 30 days	▪ Regeneration of B cells in islets of Langerhans & neoformation of islets ▪ Treatment protected against hydrophobic degeneration in liver ▪ ↑ Glycogen localization in liver [26]
**4. Lipid Regulation**	▪ C57bl6/J, high fat diet ▪ Bitter melon seed oil ▪ 5 weeks	White adipose tissue: ▪ ↑ Phosphorylation of acetyl-CoA carboxylase ▪ ↑ cAMP-activated protein kinase (PKA) [27]
	▪ C57BL/6, high fat diet ▪ Bitter melon fruit ▪ 12 weeks	Epididymal adipose tissues (EAT) and brown adipose tissues (BAT) ▪ ↓ Mast cell recruitment ▪ ↓ Interleukin-6 (IL-6) and tumor necrosis factor-α (TNF-α) ▪ ↓ Macrophage infiltration↓ M1/M2 phenotype ratio of macrophages [28]
	▪ Sprague-Dawley rats, high fat diet ▪ Bitter melon juice ▪ 7 weeks	▪ ↓ Visceral fat mass, hepatic triacylglycerol ▪ ↑ Serum free fatty acids ▪ ↑ Peroxisome proliferator-activated receptor coactivator-1 α (PGC-1-α ) [29]

## 3. Clinical Reports

The clinical potential of bitter melon has been examined in several human cell line experiments. In studies of hepatoblastoma cells (HepG2), it has been shown to be a potent inhibitor of triglyceride synthesis and ApoB secretion [30]. Similarly, momordin (a constituent of bitter melon) increases the expression of peroxisome proliferator-activated receptor (PPAR) δ mRNA in HepG2, which is important in the regulation of glucose metabolism and fatty acid storage [31]. Bitter melon juice also has been demonstrated to significantly reduce sterol regulatory element-binding protein 1c (SREBP-1c) when applied to primary preadipocytes, further highlighting the prospective benefit of this compound in glycemic control. However, direct evidence in humans has been limited to mostly anecdotal case reports and a few conflicting studies among diabetic or non-diabetic “at-risk” patients (Table 2). Accordingly, the effect of bitter melon on blood sugar levels and disease progression in pre-diabetics must be extrapolated from these studies.

**Table 2:** Summary of clinical studies on bitter melon (*Momordica charantia*) ^§^.

First Author [Ref.] (Location)	Sample	Design	Treatment	Jadad Score	Results
Ahmad [32] (Bangladesh)	100 Moderate type II diabetics	Case series (no referent group)	Aqueous homogenized suspension (single treatment), Non-standardized dose	C	↓Fasting serum glucose ( *p* < 0.001), ↓Post-prandial serum glucose (*p* < 0.001)
Akhtar [33] (Pakistan)	8 Uncomplicated, maturity-onset diabetics	Case series (no referent group)	Powdered bitter melon, 50 mg/kg body weight twice daily for 7 days after breakfast and dinner along with milk	C	↓Mean blood sugar levels (*p* < 0.05)
Baldwa [34] (India)	5 Healthy volunteers, 5 Diabetic referents, 9 Pts. with diabetes mellitus	Unblinded, Non-randomized, Clinical trial	Subcutaneous (single injection) of Polypepide-p, Arbitrary dosing	C	↓Fasting serum glucose (*p* < 0.05)
Dans [35] (Philippines)	40 Newly physician diagnosed or poorly controlled type 2 diabetes	Double-blind, Randomized, Placebo-controlled, Intent-to-treat	2 Capsules of bitter melon thrice daily for 3 months, Medication prepared by 3rd party to conceal allocation	B	↓Fasting plasma glucose ( *p* = 0.5862), Achieved study power was only 11%
Fuangchan [36] (Thailand)	143 Newly diagnosed type 2 diabetes	Double-blind, Active-control, Pill counting to monitor compliance , Intent-to-treat	2 Capsules (500 mg) before and after meals for 4 weeks	A	↓Mean fructosamine levels (−10.2 µmol/L; 95%CI = −19.1 to −1.3)
Grover [37] (India)	14 NIDDM (mixed sex), 6 IDDM (female only)	Case series (no referent group)	Bitter melon seeds, Single treatment	C	↓Post-prandial serum glucose (*p* = 0.001)
Habib [38] (Bangladesh)	8 NIDDM (3F, 5M) taking ½–1 tablet glibenclamide randomly selected from diabetic center	Open-label, Cross-over design (15 day wash-out period between treatments)	Powdered, dried fruit (4 gm/patient) administered consecutively for 21 days	C	↓Post-prandial serum glucose ( *p* = 0.001)
John [39] (India)	26 Type 2 diabetics	Unblinded, Randomized, Clinical trial	2 Tablets (1 gm) dried fruit thrice daily after meals for 4 weeks	C	↓Fasting serum glucose ( *p* > 0.05), ↓Post-prandial serum glucose (*p* > 0.05)
Kasbia [14] (Canada)	5 Non-diabetic, overweight men	Randomized, Blinded, Placebo-controlled, Cross-over study	Eligible participants underwent 3 experimental conditions in a randomized order: placebo, 50 mg/kg,100 mg/kg body weight of freeze-dried juice, Single treatments separated by 1-week washout period, Placebo capsules filled with inert cellulose	C	No effect on plasma glucose
Khanna [40] (India)	6 Juvenile referents, 5 Juvenile diabetes, 2 Maturity onset diabetes referents, 6 Maturity onset diabetes	Unblinded, Non-randomized, Clinical trial	Subcutaneous, Polypepide-p (single treatment), Arbitrary dosing depending upon severity of diabetes	C	↓Serum glucose ( *p* < 0.05) at selected time point
Kochhar [41] (India)	60 Non-insulin-dependent male diabetics	Case series (no referent group)	Raw powered mixture (bitter melon fruit, fenugreek seeds, jambu seeds) in the form of capsules (Group I) or salty biscuits (Group 2), Daily supplementation of 1 g for 1.5 months followed by 2 g for 1.5 months, Fresh, immature bitter melon fruit procured from a single lot grown locally and stored in airtight plastic containers until use	C	↓Fasting glucose ( *p* < 0.01), ↓Postprandial glucose (*p* < 0.01), ↓Decreased oral hypoglycemic drug intake (*p* < 0.05)
Leatherdale [42] (England)	9 NIDDM	Case series (no referent group)	Water-soluble extract of bitter melon fruit (single treatment), Later received fried bitter melon fruit for 8 to 11 weeks	C	↓Blood glucose concentration during 50 g oral glucose tolerance test ( *p* < 0.05)
Lim [43] (Philippines)	40 Newly diagnosed type 2 diabetes mellitus (18 males, 22 females)	Double-blind, placebo-controlled trial, Medications prepared by a third party to conceal allocation	Tablets containing dried bitter melon leaves, Single oral dose (3 treatment groups of 60, 80, 100 mg/kg/day), Placebo tablets matched in appearance to active compound	B/A(published in journal without impact factor or indexed in PubMed)	↑Insulin levels at 15 min ( *p* = 0.0402), ↓Average plasma glucose levels at 15 min (*p* = 0.0121), 100 mg/kg/day dose of bitter melon was more effective than lower doses and placebo in reducing mealtime glycemic excursions within 4 hour postdose, with more rapid return to baseline levels
Pons [44] (Puerto Rico)	8 Patients (7 diabetic, 1 normal)	Case series (no referent group)	2 Pills (0.10g of bitter melon extract powder) taken after each meal, Duration of administration varied up to 3 weeks	C	↓Blood sugar in 2 patients ( *p* > 0.05)
Purification, (unpublished report in Duque [45]) (Phillipines)	260 Type 2 diabetics	Phase III trial, Randomized block design, (bitter melon, n = 128; glibenclamide, n = 132)	Tablet (100 mg/kg/day) for 12 weeks	C (sufficient details not provided)	↓Fasting plasma glucose ( *p* < 0.05), Bitter melon and glibenclamide were comparable in terms of efficacy and safety
Rosales [46] (Philippines)	27 Type 2 diabetics with suboptimal glycemic control	Unblinded, Randomized, Cross-over, Clinical trial	200mL bitter melon tea after meals, Placebo consisted of tea made from leaves of *Camellia sinensis*, 24 Week study	C	↓HbA1c (63% reduction, *p* = 0.005), ↓Fasting serum glucose (*p* = 0.403)
Srivastava [47] (India)	7 Mild to severe diabetics	Case series (no referent group)	100 mL Aqueous extract, Separate group given 15 g dried fruit powder thrice a day in equal doses of 5 g each, 3 Week treatment period	C	↓Post-prandial serum glucose for aqueous group only ( *p* < 0.01)
Tongia [48] (India)	15 NIDDM	Unblinded, Non-random allocation	200mg Twice daily soft bitter melon extract (7 days treatment),	C	↓Post-prandial serum glucose ( *p* < 0.001)
Tsai [49] (Taiwan)	42 Patients with metabolic syndrome (mean age 45.7; range 23 to 63 years)	Open-label, Uncontrolled, Supplementation trial	4.8 g Lyophilized bitter melon powder daily for 3 months	C	↓Metabolic syndrome ( *p* = 0.021), Improved insulin resistance at visit 2 and return to nearly baseline levels after stopping bitter melon (*p* > 0.05)
Welhinda [50] (Sri Lanka)	18 Newly diagnosed maturity onset diabetics	Cross-over design	100 mL Aliquots of clear bitter melon juice given orally, Placebo (same patients) received 100 mL distilled water, Single administration of treatment and placebo	C	↓ Glucose tolerance curves compared with referent glucose tolerance curves ( *p* < 0.01)
Zänker [51] (Germany)	124 Type 2 diabetes mellitus (age > 40 years) not treated with insulin but receiving anti-diabetics	Prospective, placebo-controlled, randomized, double-blinded, Participants required to stop taking dietary supplements, Compliance not monitored	Special water soluble bitter melon standardized at 10% charantin, Daily dose - 1 g (2 capsules), Second active group received chromium and zinc supplementation, Screening phase + 4-week depletion phase + 4-month intervention phase, No information provided on the country of origin for bitter melon, part of the plant used (e.g., seeds, pulp, leaves), or self-life	B/A (published in journal without impact factor or indexed in PubMed)	↓ HbA1c, *p* = 0.0002, No hypoglycemic or other adverse events reported

^§^ Studies identified through a search of PubMed, Google Scholar, and published manuscript reference lists using key words (*Momordica charantia*, bittermelon, bitter gourd, and karela). A ≥ 4, B = 2–3, C ≤ 1; NIDDM = non-insulin dependent diabetes mellitus; IDDM = insulin dependent diabetes mellitus.

Studies illustrating the benefit of bitter melon on lowering blood sugar have appeared sporadically in the literature for over 70 years. On the island countries of Puerto Rico, Cuba, and the Dominican Republic, bitter melon has been traditionally used to treat high blood sugar and diabetes since the early 1900s [44]. In a non-randomized administration of bitter melon in the form of a dried powder alcoholic extract to eight patients (seven diabetic, one normal) at a clinic in San Juan, Puerto Rico, a positive blood sugar lowering effect was observed in two of the patients, one of whom was non-diabetic [44]. However, it was emphasized in this report that “the continued and indiscriminate use of any such remedy by the public, if ineffective, unavoidably results in detrimental and even fatal procrastination of appropriate treatments.” 

In Britain, immigrant herbal practitioners have long claimed that bitter melon (karela) is an effective therapy for lowering blood sugar levels [52]. In one of the first case reports to be published in a main-stream medical journal, bitter melon reportedly reduced the urine sugar levels of a 40 year-old diabetic Pakistani woman, who hitherto had difficulty controlling her diabetes with conventional drug (chlorpropamide) therapy and dietary restriction [52]. Furthermore, she was able to effectively reduce the dose of chlorpropamide necessary to control her high blood sugar. However, physicians were cautioned to be aware of this potential food-drug interaction among their Indian and Pakistani diabetic patients who also may be receiving concurrent treatment from an herbal practitioner. 

Polypeptide-p is one of the few active compounds in bitter melon that has been studied in clinical trials. The extraction of polypeptide-p (consisting of 166 residues) from the fruit, seeds, and tissue of frozen bitter melon was first described at the Third International Congress of Plant Tissue and Cell Culture held in Leicester, England during the summer of 1974 [34]. The compound closely resembles bovine insulin with the exception of one extra amino acid, methionine [40]. Sometimes referred to as “plant insulin”, polypeptide-p tests negative in immunoassays against bovine insulin and reportedly does not invoke an antigenic response in humans. Clinical research examining the effect of polypeptide-p on blood sugar levels has mainly focused on the subcutaneous administration of this compound. By circumventing the neutralizing proteolytic enzymes of the intestines, polypeptide-p is believed to have greater activity when injected subcutaneously compared with oral administration [13]. 

In a study of nine patients with diabetes mellitus (six juvenile, two asymtomatic, one maturity onset) ranging from age 16 to 52 years, subcutaneous injection of polypeptide-p resulted in a statistically significant drop in mean blood sugar levels (2-tailed, paired *t*-test, *p* = 0.05) [34]. No appreciable decrease in blood sugar levels was observed among either normal (n = 5) or diabetic (n = 5) referents receiving placebo injections. However, participants were not randomized between bitter melon and placebo. Compared with a typical peak effect seen after 2–3 h for regular insulin, polypeptide-p treated patients peaked after 4–12 h. A similar hypoglycemic effect was observed in a subsequent (non-randomized) study (presented at the 20th Annual Meeting of the American Society of Pharmacognosy, Purdue University) in which polypeptide-p was subcutaneously injected into 11 diabetic patients (five juvenile, six maturity onset) compared with eight diabetic referents (six juvenile, two maturity onset) [22]. Those with a history of ketoacidosis, cerebrovascular accidents, acute myocardial infarction, and renal failure were excluded from the study. 

Among non-insulin-dependent diabetics, a water-soluble extract from locally obtained fresh (raw) bitter melon juice has been observed to significantly reduce mean blood sugar levels and correspondingly increase mean insulin levels during a 50 g oral glucose test, compared with standard test of distilled water (*p* < 0.05) [42]. Frozen juice squeezed from bitter melon pulp and seeds from the plant manifested equally positive results in studies of diabetics [37,50]. However, a weaker effect upon glucose levels was reported following the consumption of either fried fruit or dried powder forms of bitter melon [33,42]. A tea prepared from bitter melon leaves reduced HbA1c levels by 63% (*p* = 0.005) compared with a referent tea (*Camellia Sinensis*) [46]. Anecdotally, some of the patients who received bitter melon powder for 7 days claimed to remain well and complication-free without any medication for about 1–2 months [33]. In a preliminary open-label uncontrolled supplementation trial of 42 participants, 4.8 g of lyophilized wild bitter melon powder significantly decreased the rate of metabolic syndrome after three months of supplementation and the improved status remained for one month after the supplementation ceased (*p* = 0.021) [49]. An increase of insulin resistance also was noted for visit 2, but did not reach statistical significance. In a small case series of seven patients, an aqueous extract of 100 g of chopped bitter melon fruit taken each morning for 3 weeks resulted in a 54% drop in blood sugar levels (*p* < 0.01) [47]. The effect was much lower (25% drop) in the powder-treated group. Furthermore, drinking an aqueous suspension of bitter melon pulp led to decreased levels of both fasting and post-prandial blood sugar (mean reduction of 18%) in a case series of 100 moderate non-insulin dependent diabetics [32]. 

A statistically significant decrease (*p* < 0.001) in post-prandial blood sugar level was observed among patients administered 4 g of bitter melon fruit powder for 21 days in a cross-over study of 8 non-insulin dependent diabetic patients (five males, three females already taking ½–1 tablets daily of glibenclamide) randomly selected from the diabetic center in Rajshahi, Bangladesh [38]. Patients ranged in age from 35 to 60 years. Each was served the same breakfast items before a 5 mL blood sample was taken 2 h later. A combination regimen of bitter melon and fenugreek seeds was found to have a synergistic effect in lowering blood sugar levels. In a similar study, a powdered mixture of bitter melon fruit, jamun seeds, and fenugreek seeks administered to 60 non-insulin-dependent male diabetics significantly reduced their fasting and postprandial glucose levels and decreased their dose of oral hypoglycemic drugs [41].

Only a few randomized controlled trials of bitter melon have been conducted. Study designs differed and a meta-analysis pooling results was not performed. A statistically significant mean decrease in fructosamine, following a 4-week regimen of 2,000 mg/day of bitter melon, compared with baseline levels, was reported in a clinical trial (randomization to 4 groups, intention-to-treat design) of patients with newly diagnosed type 2 diabetes conducted at four hospitals in Thailand (−10.2; 95% confidence interval=−19.1–1.3 µmol/L) [36]. Roasted rice powder (which has a high glycemic load) was used as the placebo comparison. Paradoxically, bitter melon had no apparent effect on fasting glucose or 2-hour glucose tolerance measurements. Three other groups were tested in this study and it is not clear if the statistical significance of the above finding holds after adjustment for multiplicity. In another positive randomized clinical study (n = 15 noninsulin dependent diabetics), bitter melon was observed to act in synergy with two oral hypoglycemics (metformin, glibenclamide) [48]. After 7 days of treatment, greater than full dose efficacy (10% to 21% reduced sugar levels) was seen for bitter melon plus half doses of metformin and glibenclamide, illustrating the potentiated effect of the combined administration in diabetics. However, residual confounding, given the small sample of the study, could have biased the results. 

No statistically significant change in serum fructosamine over time (baseline to 4 weeks of treatment) was observed for bitter melon (6,000 mg/day) compared with placebo (riboflavin) in a randomized controlled clinical trial (treatment n = 26; Placebo n = 24) [39]. While a mean drop in fructosamine from 350.8 to 319.1 mg/dL was observed for the bitter melon arm from baseline to 4 weeks, an unexplained drop in the placebo group also was observed over the same time period (349.1 to 333.9 mg/dL). Details were not provided regarding the standardization of bitter melon or whether patients complied with their routine oral hypoglycemic agents. Similarly, the mean beneficial change in fasting blood sugar levels from pre- to post-treatment (3 months, mean decrease = 0.41 mmol/L) with bitter melon was not statistically significant in a randomized, double-blind, placebo-controlled trial conducted at outpatient clinics in the Philippines [35]. However, the achieved power for this study was only 11%. Another (non-peer reviewed) study, conducted over a 10-year period by the Philippine Council for Health Research and Development (PCHRD) reported that a 100-milligram per kilo dose per day of bitter melon was clinically comparable to 2.5 mg of the anti-diabetic drug glibenclamide taken twice per day [45,53] for regulating blood sugar [45]. A pilot study conducted in five non-diabetic overweight men did not observe any effect on plasma glucose levels after they consumed a freeze-dried juice extract (100 mg/kg body weight) of bitter melon [14]. 

Recently, a prospective, randomized, double-blinded and placebo-controlled trial of bitter melon reported a statistically significance decrease in HbA1c levels after 4 mouths of intervention, compared with a referent group receiving refined soybean oil [51]. In this study, bitter melon was administered as a special water soluble power (2 g daily dose), standardized at 10% charantin. Bitter melon was well-tolerated and no apparent differences in liver function values were observed in comparison with the referent group. In a study of 40 newly diagnosed patients with type 2 diabetes mellitus (18 males, 22 females), a single dose (100 mg/kg/day) of bitter melon was observed to be significantly more effective than those randomized to placebo (double-blind) in reducing mealtime glycemic excursions (within post-dose window) and reverting more rapidly to baseline levels [43].

Alone, few side effects have been associated with the use of bitter melon. No deaths have been reported to date. The most commonly observed adverse effects include mild diarrhea and abdominal pain, which subside after discontinuing use [54]. However, bitter melon has been shown to potentiate the effect of certain drugs used to treat diabetes, possibly resulting in hypoglycemia [52]. Rare cases of hypoglycemic coma and convulsions have been reported in children drinking bitter melon tea [55]. A single case report has suggested that bitter melon may induce paroxysmal atrial fibrillation, based on a Naranjo adverse drug reaction score of 6 (corresponding to a probable causal association) [56]. Bitter melon use also is contraindicated during pregnancy because of its abortifacient properties [57]. The risk associated with the long-term use of bitter melon has not been studied.

## 4. Synthesis of the Literature

Differences in the effectiveness observed across studies examining the potential glucose lowering effects of bitter melon may be attributable to several factors. Ayurvedic physicians typically insist on the daily preparation of herbal diabetic remedies intended for oral consumption [50]. The aqueous extract of Aegle Marmelos, a herb reputed to have oral hypoglycemic activity similar to bitter melon, reportedly loses 66% of its maximum hypoglycemic potency after just 72 h of room temperature storage [50]. The decline in potency is believed to be due to either bacterial activity or other yet-to-be understood explanations [50]. Factors potentially influencing bitter melon efficacy include differences in soil/climate, harvest time, part of the plant used (e.g., leaves are believed to be more effective), and how the plant is cooked and prepared (e.g., over-heating, the addition of other ingredients that may affect absorption). Different variants of bitter melon (shape, color, size) also may be found throughout the world and could affect the biologic activity of the herb [58]. Information on the above factors typically was not collected in the studies considered for this review. Furthermore, studies often lacked a population-based referent group, were not adequately blinded, failed to conceal allocation assignment, were too short in duration to yield effect, or suffered from selection bias (not randomized) [36]. Such biases tend to be associated with inflated study benefits [59,60]. In other cases, statistical methodology, design details, and study conduct (e.g., inclusion/exclusion criteria, ethnic background of patients, drop-out rates, *a priori* power analysis, baseline comparability, outcome measures, and treatment adherence/integrity) were not adequately described to allow a definitive comparison across studies or a basic assessment of face validity [61,62]. Among the manuscripts reviewed in Table 2, less than 20% received a quality score of two or greater on the Jadad scale [62].

While bitter melon holds promise for the safe and affordable prevention and control of high blood sugar among pre-diabetics, primary care providers must err on the side of caution and appropriately advise patients not to forego medical consultation and conventional therapy (especially as their disease progresses) in lieu of anecdotal evidence or recommendation from traditional herbal practitioners. Patients with poor glycemic control, cardiovascular disease, fatty liver disease, and polycystic ovary syndrome must be closely monitored and queried regarding their use of bitter melon and other herbal products that may interact with conventional medicines such as metformin and acarbose [63,64]. Furthermore, drugs approved by the Food and Drug Administration (FDA) that increase insulin sensitivity (e.g., pioglitazone) may help reduce the risk of converting from pre-diabetes to diabetes, without the uncertainty in dosing and potential for interaction associated with the uncontrolled use of bitter melon. Bariatric surgery also remains a proven intervention to control the progression to diabetes [2].

Nevertheless, the use of bitter melon may be warranted in certain individuals, such as patients without health insurance, those living in rural areas far from health care, members of ethnic/religious groups who do not subscribe to conventional medical care or surgery, and non-compliant patients (e.g., those with difficulty swallowing pills). An increasing prevalence of pre-diabetics has been noted in remote, rural regions, especially those with a high number of elderly residents [65]. Further assessment of the benefits and risks of bitter melon use in these groups is recommended.

Reducing healthcare costs also may be a factor prompting the use of bitter melon and other alternative therapies aimed at preventing and controlling high blood sugar. An advantage of medicinal plants such as bitter melon is that they may be inexpensively cultivated in gardens and greenhouses. However, potential short-term savings must be weighed in light of the effectiveness of bitter melon (or lack thereof) and the proclivity of patients to delay using clinically proven therapies. Additionally, the acceptance of bitter melon may be limited due to its undesirable bitter taste [66]. Research needs to be directed toward strategies for improving the palatability and consumption intentions of food dishes containing bitter melon.

## 5. Conclusions

Overall, it remains controversial whether bitter melon has proven benefits in lowering blood sugar among pre-diabetics or aids in slowing the progression to diabetes. While the evidence to date, when examined as a whole, is suggestive of a possible beneficial effect, future clinical studies that meet rigorous methodological standards are warranted before attempts to establish policy recommendations regarding the use of this herb among pre-diabetics. 
